# Application of fluorescein sodium in the resection of vermis pilocytic astrocytomas

**DOI:** 10.1186/s12957-017-1108-5

**Published:** 2017-02-14

**Authors:** Ji Zhang, Fuad AL-Nahari, Zi-feng Wang, Fu-hua Lin, Yi-yin Zhao, Shi-yin Xiao, Jian-min Liu, Chao Ke, Zheng-he Chen, Yu Jiang, Men Yang, Ke Sai, Jian Wang, Yong-gao Mou

**Affiliations:** 1 Department of Neurosurgery, State Key Laboratory of Oncology in South China, Sun Yat-sen University Cancer Center, Collaborative Innovation, Center for Cancer Medicine, 651 Dong Feng East Road, Guangzhou, 510060 China; 2 Department of Neurosurgery, The second affiliated hospital of Guangzhou medical university, Guangzhou, China; 3 Department of Neurosurgery, Department of Neurosurgery, The First Affiliated Hospital of Guangzhou University of Traditional Chinese Medicine, Guangzhou, China; 4 Department of Anesthesiology, State Key Laboratory of Oncology in South China, Sun Yat-sen University Cancer Center, Collaborative Innovation Center for Cancer Medicine, 651 Dong Feng East Road, Guangzhou, 510060 China; 5 Department of Thoracic surgery, state Key Laboratory of Oncology in South China, Sun Yat-sen University Cancer Center, Collaborative Innovation Center for Cancer Medicine, 651 Dong Feng East Road, Guangzhou, 510060 China

**Keywords:** Fluorescein sodium, Vermis pilocytic astrocytoma, Surgery, Microscopy

## Abstract

**Background:**

Pilocytic astrocytomas (PAs) are slow growing neoplasms and usually located at the cerebellum. There has been certainty regarding the truthful benefit of surgical resection for patients with PA. Gross total resection (GTR) of PAs, especially those being situated in deep regions, remains a surgical challenge. Generally, they are considered as benign and usually develop in young patients. PAs, belonging to WHO I can be cured by radical resection. The patients with PA have excellent prognosis if complete resection can be conducted. The use of fluorescein in vermis PA surgery has not been yet reported. Our data presents fluorescein facilitates surgical resection of vermis PA.

**Methods:**

Five milligrams per kilogram of fluorescein sodium was intravenously injected directly before general anesthesia for the three patients with PA. The yellow 560 filter was employed for microsurgical tumor resection. Surgical outcomes were assessed concerning the extent of resection.

**Results:**

Most portion of PA in the three cases was found to be highly fluorescent after intravenous fluorescein sodium injection, which markedly enhanced tumor visibility. Gross total resection in all of the patients was achieved without further neurological deficits. No adverse effects and complications resulting from fluorescein sodium were observed over the postoperative course.

**Conclusions:**

Intraoperative guidance by fluorescein sodium as a new, simple, safe, and practical procedure can enhance the fidelity of tumor tissue and increase the possibility of completely resecting PAs.

## Background

Pilocytic astrocytomas (PA) is a unique subtype of glioma in clinical behavior [1]. A large number of clinical studies have repeatedly underlined the value of surgical treatment for patients with PAs [2]. The extent of resection had a powerful influence on the progression-free survival (PFS). The fact that has been constantly shown to be crucial for overall survival (OS) and PFS is that patients benefit complete resection of PAs.^3^ Complete resection is a goal of tumor removal during open surgery. Fluorescein sodium can make tumor tissue appear yellowish, which can be visualized against a normal background. This can help to identify the target and thus facilitate total tumor removal under microscopy. To the best of my knowledge, the use of fluorescein sodium in vermis PAs’ surgery has not been yet reported.

## Methods

Informed consents were obtained from patients with vermis PAs about the use of fluorescein sodium. Three patients had been surgically treated at our neurosurgical department. Comprehensive data including preoperative and postoperative MRI scans within 72 h was documented. Surgery was performed with a surgical microscope equipped with a yellow 560 filter. A predefined dose of 5 mg/kg of fluorescein sodium was intravenously injected via a central line immediately before general anesthesia. All patients were admitted to the neurosurgical common care unit for postoperative care. Postoperative MRI images were reviewed for any contrast-enhancing residual tumor tissues by a neuroradiologist and neurosurgeon. The presence of PAs was confirmed in all patients by a neuropathologist. The surgical reports were screened with reference to the degree of fluorescent sodium staining: “bright/helpful” versus “effectively no fluorescence/not helpful”. The study was approved by the local ethics committee.

### Radiological examination

**Fig. 1:**
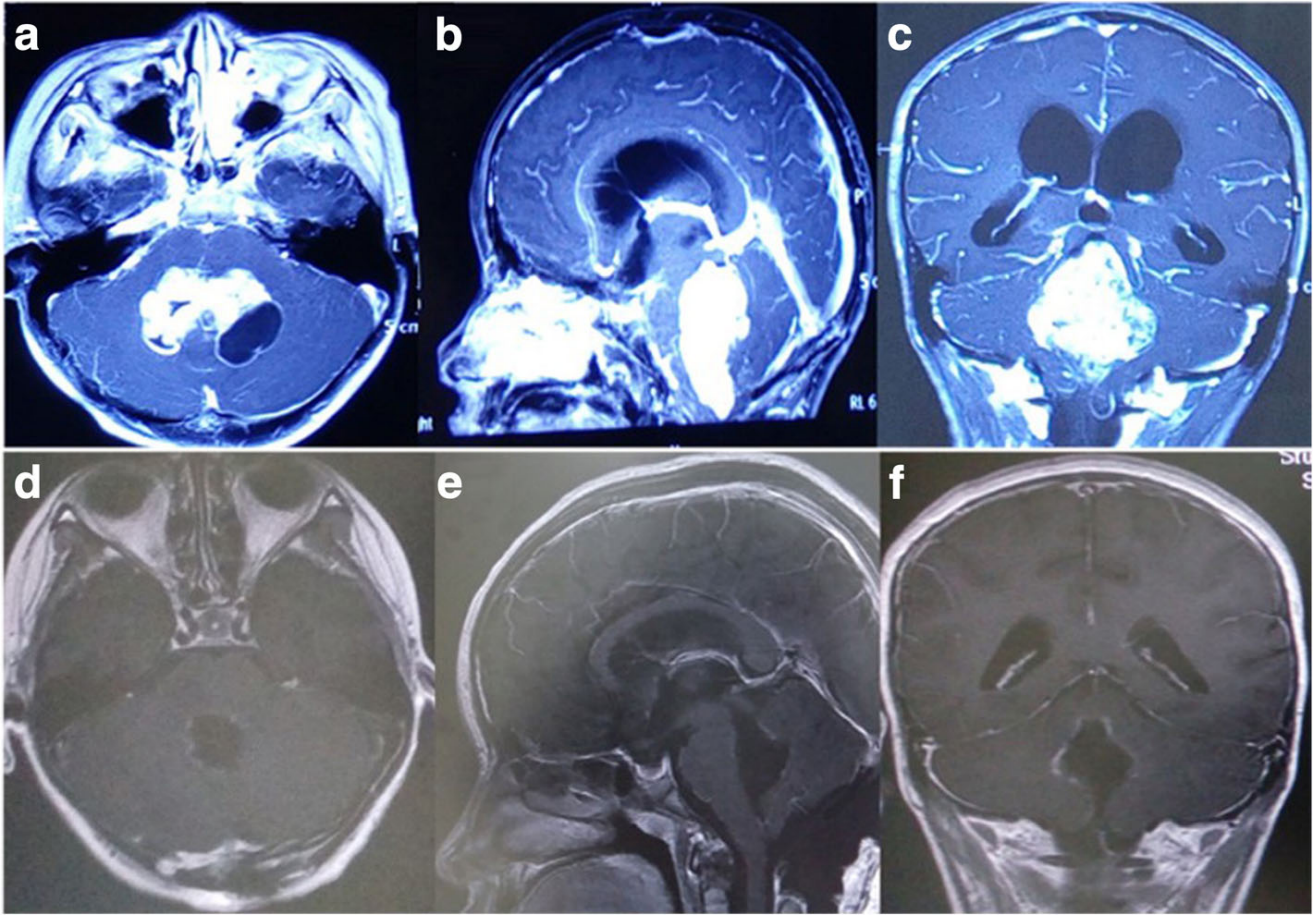
Preoperative axial (**a**), sagittal (**b**), and coronal (**c**) MRI illustrating a large mass with an enhancing solid mass and associated cyst in the fourth ventricle. Postoperative MRI (**d**–**f**) revealing complete removal of the tumor

Vermis PAs, accounting for a low rate in the posterior fossa PAs, are commonly hypodense on precontrast CT scans and hyperdense on precontrast MRIs. Vermis PAs usually do not have radiological features found with cerebellar PAs, which have characteristically well-circumscribed lesions demonstrating a mural nodule with a unique or multiple macrocysts. The solid portion obviously demonstrates homogeneous contrast enhancement. Vermis PAs are inclined to have less cystic components, with significant enhancements such as imaging features found in medulloblastomas and ependymomas (Fig. 1a–c). Our goal was to evaluate the extent of resection under the aid of fluorescein sodium in the three patients with vermis PAs. Postoperative MRI was obtained within 72 h for the assessment of residual tumor. Tumor gross total resection (GTR) was defined as no residual enhanced tumor on the postoperative Gd-enhanced T1-weighted MRI (Fig. 1d–f).

### Surgical procedure

**Fig. 2:**
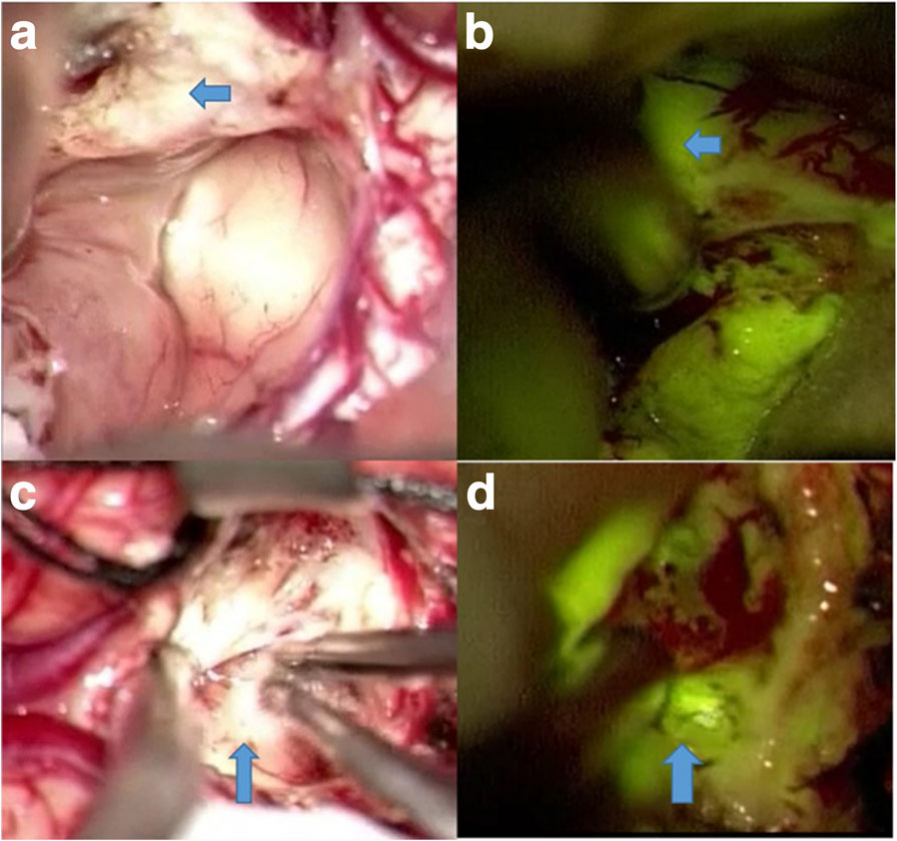
Microsurgical photograph of the vermis under white light (**a**, **c**) and the yellow 560 filter (**b**, **d**) from intermediate stages of tumor resection. Residual tumor tissue (*arrow*) was identified under the yellow 560 filter compared with white light (**b**, **d**)

**Fig. 3:**
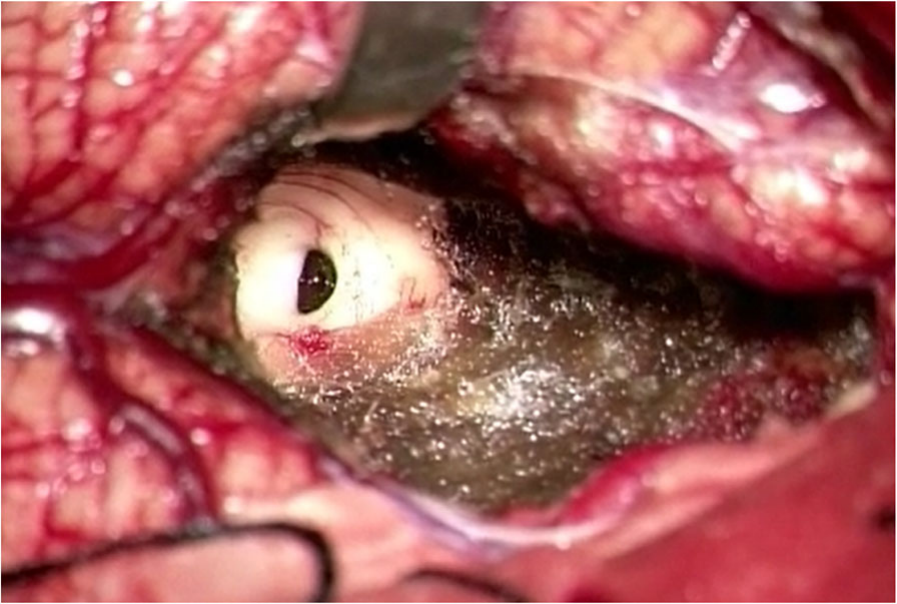
Intraoperative microsurgical photograph displaying total resection of the tumor and the exit of the midbrain aqueduct

Fluorescein sodium (5 mg/kg) prior to general anesthesia was administered into the central venous line. Craniotomy was performed conventionally. Following the administration of fluorescein sodium, fluorescence could be visualized by utilizing a yellow fluorescence microscope system. The resection was executed approximately 1 h after administration of fluorescein sodium with the aid of white light in conjunction with intraoperative fluorescence guidance (Fig. 2). In the three cases, tumors were removed piece by piece based on fluorescein identification. Resection was continued until no highly visible fluorescent tissue remained (Fig. 3).

## Results

High fluorescent staining of the lesion was documented, which markedly enhanced tumor visibility. We decided to remove all fluorescent tissue as tumor cells could not be excluded during frozen section. Pale fluorescence or absence of fluorescence staining of the tumor had not been observed in our cases. Fluorescein staining provided intraoperative identification of residual tumor tissue, which is confirmed by the data presented here. The case presentation was chosen to display the impression of fluorescence: the views under the white light (Fig 2a, c) do not explicitly show a lesion, but the views under the yellow filter (Fig. 2b, d) depict the fluorescent signal that clearly demarcates the targeted area. No patients showed significant residual tumor tissue upon postoperative MRI examination. We did not observe any adverse or allergic reactions or disorders related to the administration of fluorescein sodium.

## Discussion

PAs, which are tending to arise in the cerebellum, are a distinct histologic and biologic subset of gliomas [1]. When diagnoses are made, most of the patients have clinical signs of cerebellar dysfunction, such as appendicular dysmetria, truncal steadiness, decreased vision, extraocular movement abnormalities, dysmetria, and gait unsteadiness [2]. These tumors easily give rise to hydrocephalus, followed by increased intracranial pressure. It is well known that the prognosis of PA is evidently associated with radical resection. The prognosis of partially extirpated PA is not satisfactory despite subsequent treatment consisting of radiotherapy and chemotherapy [3]. In order to increase the resection rate of giant vermis PAs, we used yellow fluorescein techniques during surgery.

The surgical management of PAs has long been a subject of affirmation. Patients with PAs have the best overall survival if the tumor is completely resected [4]. Complete removal is associated with a 10-year survival rate of 100% and a low recurrence rate, whereas almost half of the patients with partially removed PAs will have recurrence [5]. Malignant transformation after partial resection due to residual tumor tissue has been constantly mentioned in the published literatures due to residual tumor tissue [6, 7]. An accurate diagnosis may not be granted if the partial removal does not include the malignant portion of the tumor [8]. Thus, radical resection to forestall recurrence is essential [9, 10].

Intraoperative use of fluorescein sodium can help to enhance the fidelity of tumor tissue, and enable complete resection of vermis PAs. With the development of novel surgical tools, the surgical resection rate has increasingly risen [11, 12]. Methods to differentiate tumors from normal brain tissue are still being sought. Substances such as fluorescein can be employed for intraoperative visualization of tumor tissue [13]. Fluorescein can permeate damaged areas of the blood-brain barrier, which are representative of tumor tissue, and result in effective and easier resection. This minimizes normal tissue manipulation and facilitates microsurgical dissection of the lesion from the surrounding parenchyma. Due to their normal structures, it is important to identify the margin between normal tissue and tumor. Fluorescein sodium can aid in differentiation so that permanent deficit due to surgical manipulation can be avoided.

PAs are generally well-circumscribed tumors that have a low growth rate. On imaging, they often present as a small solid nodule accompanied by a big cyst [14]. The borders of vermis PAs with less cystic elements in our series of patients were not clear under microscopy. Some studies reported that fluorescein sodium reduced surgical difficulty and increased resection rate compared to studies where it was not used. Fluorescence-guided surgery is an easy procedure and does not require additional time and instrumentation. Utilizing a microsurgical mirror, identification of residual fluorescent tissue within the resection cavity facilitates complete resection and can be verified on postoperative MRI. In the current study, the data suggests that the use of FL was safer and increased resection of vermis PAs compared to simple standard microsurgery.

The mean duration of surgery for the fluorescein group was shorter than that of the standard microsurgery group. Complete resection of contrast-enhanced tumor tissue has been repeatedly indicated to be crucial for lowering recurrence rate, PFS, and the length and quality of survival [15]. Despite the limited number of patients in our data, this study illustrated that fluorescein facilitated complete removal of vermis PAs. Here, we rate a rate of 100% for positive fluorescein staining under the yellow 560 nm filter. Absence or insufficient tumor fluorescence staining during resection was not observed in our three patients.

Fluorescein has been increasingly reported to obviously improve the efficacy of fluorescence-guided resection of malignant gliomas [16–18]. Fluorescein gathers in a disrupted blood-brain barrier area as a result of pathologically increased vascular permeability [19, 20]. We injected a low dose of fluorescein sodium (5 mg/kg) and used the dedicated surgical microscope with a yellow 560 nm filter. It was relatively easy to visualize the discretely fluorescein-stained tumor tissue. The visible fluorescence effect accurately confirmed the tumor locations through pathology and resulted in decreased surgical damage to brain tissue surrounding the tumor. Bright fluorescence signaling was observed in all patients, which aided in the identification of a suspicious lesion. Accordingly, the application of fluorescein sodium for contrast-enhancing vermis PAs could confirm the surgical margins and allow for safe and thorough resection. To the best of our knowledge, our group has provided the first data on the efficacy of fluorescein sodium for the resection of vermis PAs. None of the patients reported any systemic or local side-effects. Any adverse effects or anaphylactic disorders were not observed.

The content of this study surpassed that of a simple feasibility study for the vermis PAs. We are well aware of the limitations of our study; namely, this study is retrospective and has no qualitative statement regarding fluorescent staining in the vermis PAs.

## Conclusions

Our clinical data illustrates the safety and utility of fluorescein sodium in the identification and resection of vermis PAs. The application of fluorescein sodium was a well-tolerated procedure that allowed for a greater extension of resection and a significantly reduced permanent deficit rate owing to the identification of crucial structures. A long-term follow up with a larger number of patients is necessary to establish the significance of fluorescein sodium-guided surgery for patients with vermis PAs.

## Abbreviations

GTR: Gross total resection
OS: Overall survival
PA: Pilocytic astrocytoma
PFS: Progression-free survival
